# Potential of Artificial Intelligence for Bone Age Assessment in Iranian Children and Adolescents: An Exploratory Study

**DOI:** 10.34172/aim.32070

**Published:** 2025-04-01

**Authors:** Mehrzad Lotfi, Nahid Abolpour, Mohammadreza Ghasemi, Hajar Heydari, Reza Pourghayumi

**Affiliations:** ^1^Department of Radiology, Medical Imaging Research Center, Shiraz University of Medical Sciences, Shiraz, Iran; ^2^Department of Artificial Intelligence, Shiraz University of Medical Sciences, Shiraz, Iran; ^3^School of Medicine, Shahid Beheshti University of Medical Sciences, Tehran, Iran

**Keywords:** Artificial intelligence, Bone age, Deep learning, Neural network

## Abstract

**Background:**

To investigate whether the bone age (BA) of Iranian children could be accurately assessed via an artificial intelligence (AI) system. Accurate assessment of skeletal maturity is crucial for diagnosing and treating various musculoskeletal disorders, and is traditionally achieved through manual comparison with the Greulich-Pyle atlas. This process, however, is subjective and time-consuming. Recent advances in deep learning offer more efficient and consistent BA evaluations.

**Methods:**

From left-hand radiographs of children aged 1–18 years who presented to a tertiary research hospital, 555 radiographs (220 boys and 335 girls) were collected. The reference BA was determined via the Greulich and Pyle (GP) method by two radiologists in consensus. The BA was then estimated to use a deep learning model specifically developed for this population. Model performance was evaluated using multiple metrics: Mean square error (MSE), mean absolute error (MAE), intra-class correlation coefficient (ICC), and 95% limits of agreement (LoA). Gender-specific results were analyzed separately.

**Results:**

The model demonstrated acceptable accuracy. For boys, MSE was 0.55 years, MAE was 0.59 years, ICC was 0.74, and the 95% LoA ranged from -0.8 to 1.2 years. For girls, MSE was 0.59 years, MAE was 0.61 years, ICC was 0.82, and the 95% LoA ranged from -0.6 to 1.0 years. These results indicate stronger predictive accuracy for girls compared to boys.

**Conclusion:**

Our findings demonstrate that the proposed deep learning model achieves reasonable accuracy in BA assessment, with stronger performance in girls compared to boys. However, the relatively wide 95% LoA, particularly for boys, and prediction errors at the extremes of the age range highlight the need for further refinement and validation. While the model shows potential as a supplementary tool for clinicians, future studies should focus on improving prediction accuracy, reducing variability, and validating the model on larger, more diverse datasets before considering widespread clinical implementation. Additionally, addressing edge cases and specific conditions that a human reviewer may detect but the model might overlook, will be essential for enhancing its clinical reliability.

## Introduction

 Accurate evaluation of skeletal maturity plays a crucial role in diagnosing, monitoring, and planning treatments for a variety of musculoskeletal disorders, including conditions such as endocrine disorders, abnormal growth patterns, scoliosis, and limb length discrepancies. Typically, an exact estimation of skeletal maturity is achieved through the use of a hand and wrist radiograph.^1^

 A common approach uses an atlas that was developed by Greulich and Pyle. This atlas originates from a study conducted between 1931 and 1942 in Cleveland, Ohio region, involving serial examinations of 1000 healthy boys and girls. This study, a precursor to the era of computers, could be considered a ‘big data’ experiment. The participants were predominantly of White ethnicity, were born in the United States, were of Northern European descent, and had elevated socioeconomic status. The atlas includes images selected from 100 radiographs of children matching the age and sex of the reference standard. A radiologist then engages in multiple subjective assessments, comparing the individual bones of a patient to those presented in the reference standard. The radiologist subsequently assigns a bone (skeletal) age, which denotes the chronological age at which children for whom the standards were established would achieve an equivalent level of skeletal maturity.^2^

 The atlas-based approach demands extensive human effort, as individuals need to compare patient images with reference atlas images. Furthermore, this procedure is subjective, as different individuals may interpret images differently on the basis of their personal experience and specific training perspectives. Since decisions are made on the basis of visual resemblances, the foundation for diagnostic outcomes becomes challenging to quantify. Another drawback of atlas-based manual estimation is the potential for reduced reproducibility.^3,4^

 Recent artificial intelligence (AI) advancements in bone age (BA) assessment, including models like BoneXpert, VUNO Med-Bone Age, PANDA, and BoneView, demonstrate significant ethnic variability in performance due to differences in skeletal maturation patterns and dataset biases. BoneXpert, an early traditional machine learning model, shows reduced reliability in prepubescent non-Caucasian children, underestimating BA in Asian preadolescents and overestimating in adolescents, while requiring calibration for African and Hispanic populations influenced by genetic and nutritional factors. In contrast, VUNO Med-Bone Age, which employs deep learning and analyzes carpal bones, achieves higher accuracy in East Asian populations (e.g. Korean children) by addressing earlier ossification patterns, though it struggles in Middle Eastern/Turkish cohorts where Western-trained models like BoneXpert exhibit algorithmic biases. New tools like PANDA offer continuous age estimation across a broad range, enhancing efficiency. In contrast, BoneView’s exclusion of children under three and rounding practices may result in precision gaps. These disparities largely stem from training data skewed toward Western populations, leading to poor generalization in non-Caucasian groups. For instance, models optimized for North American datasets fail to capture early carpal bone maturation in Asians or unique growth patterns in Turkish children.^5-8^

 AI-based BA assessment models face considerable challenges in accurately evaluating the Iranian population due to their reliance on Western-centric datasets and methodologies. Tools like BoneXpert and convolutional neural network (CNN)-based systems, trained predominantly on Greulich-Pyle or USC atlases, exhibit reduced accuracy in Iranian children. These inaccuracies result from ethnic disparities in skeletal maturation, such as unique metacarpal dimensions in Iranian children, which traditional models fail to capture. While emerging Iran-focused AI approaches aim to address these gaps, their generalizability remains limited by small-scale datasets and insufficient validation. These shortcomings underscore the need for localized, ethnically representative AI models trained on annotated datasets that integrate Iran’s distinct genetic, environmental (e.g. nutritional factors), and clinical contexts. Prioritizing region-specific data collection, refining algorithms for ethnic variability, and standardizing imaging practices are essential steps toward developing reliable, inclusive AI solutions for BA assessment in Iran and other underrepresented populations.^9-11^

 Therefore, the present study aimed to assess the feasibility of utilizing automated deep learning techniques based on the Greulich‒Pyle method for assessing BA in healthy Iranian children. Our hypothesis was that our deep learning model’s accuracy would be comparable to that of models developed for other nationalities.

## Materials and Methods

###  Study Design

 This was a retrospective study that utilized left-hand radiographs and medical records collected from Namazi Hospital and Shahid Faghihi Hospital in Shiraz between January 2019 and January 2022. To minimize the potential for selection bias, all eligible cases within this time frame were reviewed. A total of 568 radiographs were identified, and 555 radiographs were included after excluding patients over 18 years of age and those with incomplete data. The inclusion of all eligible cases was intended to ensure a representative dataset and reduce bias inherent in retrospective designs. This was a retrospective study in which patients’ hand X-rays and medical records from Namazi Hospital and Shahid Faghihi Hospital in Shiraz were used. This study was approved by the Institutional Review Board and Ethics Committee of Shiraz University of Medical Science (IR.SUMS.REC.1401.256). Due to the retrospective nature of the study and the use of anonymized data, the requirement for informed patient consent was waived.

###  Patient Selection and Reference Standards

**Figure 1:**
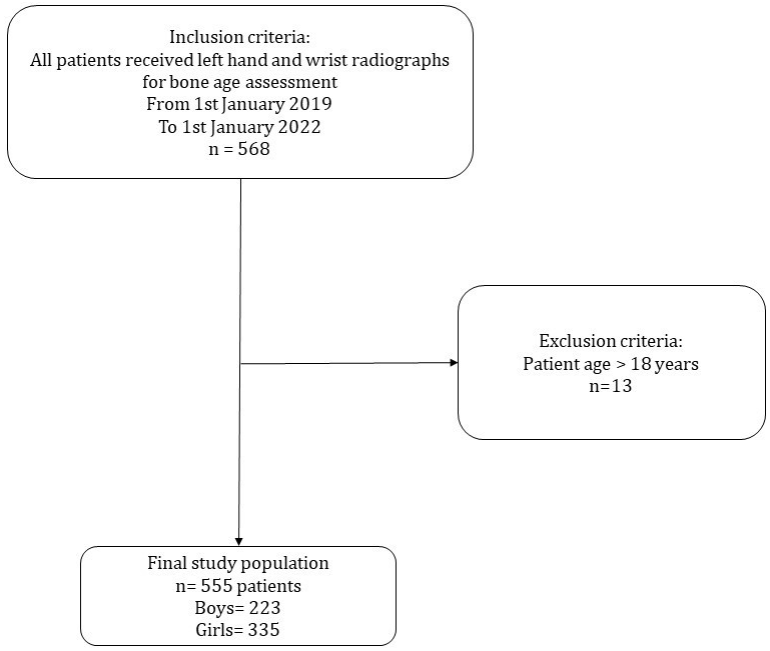
Process of Selection. Selection of study population is shown in the flowchart. A total of 573 patients with radiographs of the left hand and wrist between January 2019 and January 2022 were initially evaluated. Thirteen patients older than 18 years were excluded

 Our study included children aged 1 to 18 years who visited our hospital for growth assessment via left-hand wrist radiography between January 2019 and January 2022. The reference standard for BA assessment was established through the collaborative evaluation of two expert radiologists using the Greulich and Pyle (GP) atlas. Initially, 568 X- rays stored in the hospital’s picture archiving and communication system (PACS) were reviewed, and eligibility was determined based on the study criteria. Patients over the age of 18 were excluded, resulting in a final dataset of 555 radiographs (220 boys and 335 girls). This sample size was chosen to provide a balanced representation for assessing the performance of the AI model across genders. While sufficient for preliminary analysis, the relatively small sample size may limit the generalizability of findings to larger and more diverse populations. Conducting a power analysis in future studies would provide a more robust justification for the chosen sample size and enhance the study’s statistical rigor. In cases of disagreement during the radiograph review process, a third expert radiologist with over 30 years of experience in BA interpretation was consulted to resolve discrepancies. The selection process is illustrated in Figure 1.

###  Bone Age Assessment Model Training

 This project is aimed at detecting BA through hand-wrist radiography via AI. For this purpose, a deep neural network is employed, which uses details extracted from regions of interest (ROIs).

 In the first step, the ROIs of each image are determined via local Shannon entropy optimization. In fact, ROIs (their size is fixed at 40 × 40 pixels) are clicks whose Shannon entropy is greater than that of the other possible clicks in each image. The ROIs are automatically obtained, which are the solutions of applying a genetic algorithm in the optimization problem.

 Two important reasons can be highlighted for the first selection of these ROIs: ROIs that are clicks with maximum Shannon entropy, which are parts of the image with the most details about the age of the bone. The selection of ROIs is a primary procedure for extracting details that are highly suitable for training neural networks.

 It is not reasonable to exploit the whole image when training the neural network. Since it includes more than 240 000 pixels in each image, a large number of parameters need to be trained in that case. However, considering ROIs reduces the number of pixels to only 400 pixels for each image, which is more likely to be used for training neural networks.

 The local Shannon entropy is actually the Shannon entropy that is calculated from one click on the image. The Shannon entropy of the click is calculated as follows:


$$H=-\sum_{i=0}^{255}p_{i}\;\log\left(p_{i}\right)$$

Notably, the histogram corresponds to one click on the image.

 In the next step, these clicks or ROIs should be determined. In fact, the location of each click should be determined to maximize Shannon’s entropy.

**Figure 2:**
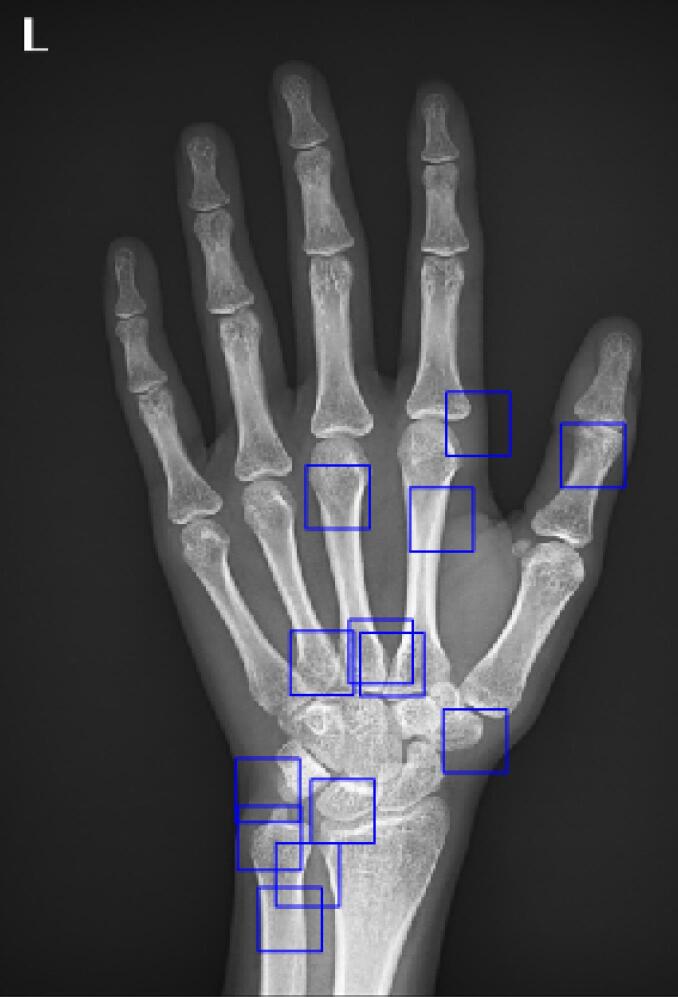
An Example of Input Image with 13 ROIs Defined

 Therefore, the location of these areas is a variable that must be determined by an optimization algorithm. In this project, the “genetic optimization algorithm” was used to determine the location of the ROIs. In this project, 13 ROIs were extracted from each image, which are shown in the following images (Figure 2):

 As stated earlier, the key regions are the clicks of the images that have the highest local Shannon entropy, and their location is determined by the genetic algorithm.

 In the next step, details are extracted from each click by specific filters. Fixed and specific filters are used in this project. Notably, in each image, we have 13 ROIs. A detail vector corresponding to each area is extracted.

 Finally, we have detailed vectors that are 13-fold the number of images, which are actually the result of applying a certain filter to the ROIs of all the images.

 Notably, for each detail vector, we have an age, which for all the details extracted from an image is a constant value.

**Figure 3:**
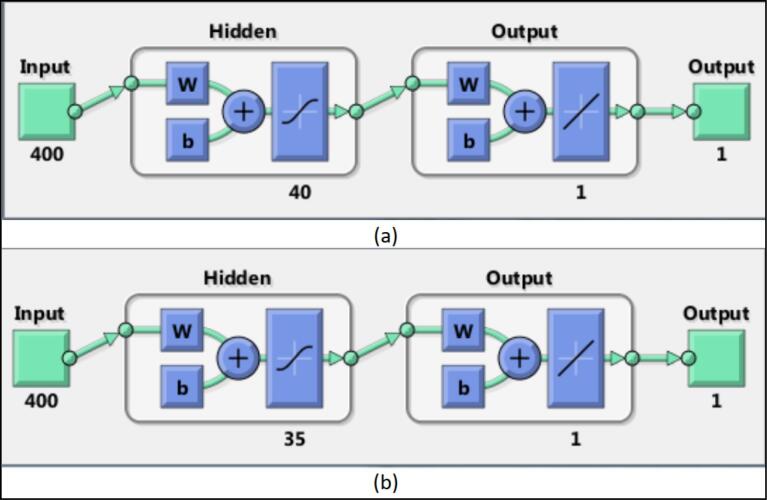
Illustration of Our Deep Learning Neural Network Architecture for Males (a) and Females (b). Number of Neurons in the First Layer are 40 for Males and 35 for Females. The letters ‘w’ and ‘b’ represent the weights and biases used in the neural network layers, respectively

 In the next step, to better train the neural network, we divided the details into two categories based on the gender of the images (boys and girls). One group of details was related to boys, and the other group to girls. The dataset was split into 70% for training, 15% for testing, and 15% for validation, following standard practices in machine learning studies. While the 70/15/15 split ensures a balanced distribution for training and evaluation, the relatively small testing and validation subsets for each gender may limit the robustness of the results. This reflects the inherent challenges of working with a smaller dataset. Nevertheless, the split was designed to optimize model training while providing independent datasets for performance evaluation. Future studies with larger datasets will help address these limitations and provide a more comprehensive assessment of model performance. Next, we trained a two-layer perceptron neural network for each category. The structure of the neural networks related to boys and girls is shown below (Figure 3).

 After determining the structure of each neural network, we train them on the basis of their corresponding image category. After training the neural networks, we measured their accuracy in detecting BA.

###  Statistical Analysis

 All statistical analyses were performed using MATLAB 15.0 and the Statistics Toolbox 15.1. The specific functions utilized included ‘fitlm’ for regression analysis, ‘corrcoef’ for calculating Pearson correlation coefficients, ‘mse’ for computing the mean square error (MSE) between the model estimates and the reference standard BAs, and ‘scatter’ for generating scatter plots to visualize the relationship between estimated and reference BAs. The overall model performance was evaluated using multiple metrics to provide a comprehensive assessment:

 Mean square error (MSE): Evaluated the average squared difference between predicted and reference BAs.

 Intra-class correlation coefficient (ICC): Assessed agreement between predicted and reference BAs, with separate analyses for boys and girls.

 95% Limits of agreement (LoA): Quantified the range within which most differences between predicted and reference BAs lie.

 Mean absolute error (MAE): Provided an alternative measure of accuracy by calculating the average absolute differences between predicted and reference values.

 Additionally, the accuracy of the estimated BAs for boys and girls was evaluated using Pearson correlation coefficients and scatter plot analysis, which demonstrated significant correlations and visually confirmed the model’s predictive capability

###  Comparison of Methods

 The deep learning model in this study employed a region-specific analysis based on the GP atlas, identifying 13 key ROIs using Shannon entropy optimization and genetic algorithms. This approach ensures that the most informative regions are prioritized for analysis, enhancing reliability by reducing noise from less relevant areas. Compared to convolutional neural networks (CNNs), which often process entire radiographs, this methodology allows for targeted evaluation and provides interpretable outputs. Interpretable AI models are increasingly emphasized in medical diagnostics to support clinical decision-making and improve trust among clinicians.

 In terms of reliability and validity, previous studies utilizing CNNs for BA assessment, such as Booz et al, Cheng et al, and Kim et al reported mean absolute differences (MADs) ranging from 0.33 to 0.65 years and ICC values between 0.70 and 0.85.^12-14^ Our model achieved comparable performance, with ICC of 0.74 for boys and 0.82 for girls and MAE of 0.59 and 0.61 years, respectively. While CNNs can achieve similar accuracy, they often lack interpretability, as they do not explicitly identify which regions of the radiograph contribute most to the prediction. This limitation can hinder clinical acceptance, particularly in cases requiring precise skeletal maturity evaluation. Additionally, other studies, such as the one by Larson et al, have demonstrated that CNNs are effective for general BA assessment but may be less robust for smaller datasets or demographic-specific populations.^15^ In contrast, our method incorporates prior anatomical knowledge and focuses on key regions, making it particularly suitable for datasets with demographic or population-specific characteristics, such as those in our study of Iranian children. This targeted approach improves clinical relevance and reduces potential biases.

 Overall, our model offers a unique balance between reliability, clinical interpretability, and validity, making it a strong candidate for integration into pediatric diagnostic workflows.

## Results

 A total of 555 BA radiographs were included in our study. All radiographs were obtained from Iranian children. The demographic information of the 555 patients is presented in Table 1. The overall mean and standard deviation of the participants’ ages were 10.75 (4.49), amounting to 11.33 (4.80) and 10.39 (4.25) for boys and girls, respectively. There were 223 boys and 335 girls. Most patients (367/555, 66.12%) were in the 6- to 14-year-old group.

**Table 1:** Age Distribution of the Dataset Image

**Age (y)**	**Boys**	**Girls**	**Total**
Total	220	335	555
0-2	1	2	3
2-3	8	10	18
3-4	13	20	33
4-5	12	8	20
5-6	4	18	22
6-7	17	16	33
7-8	2	34	36
8-9	12	35	47
9-10	3	12	15
10-11	6	24	30
11-12	23	43	66
12-13	8	41	49
13-14	31	11	42
14-15	16	20	36
15-16	15	15	30
16-17	13	8	21
17-18	10	13	23
18-19	14	17	31

**Figure 4:**
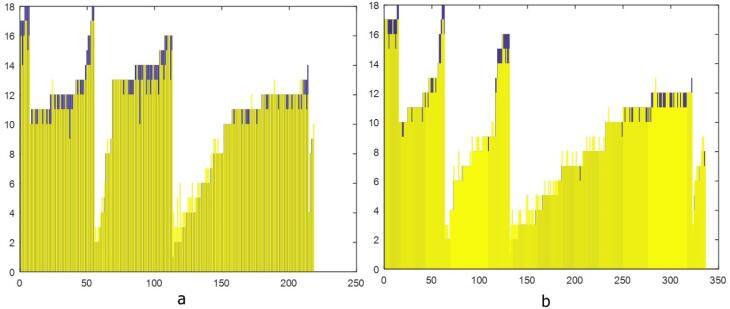
Reference Standard and AI-estimated Bone Age Values of Boys (a) and Girls (b)

 The following chart illustrates the reference standard and estimated BA values for boys and girls (Figure 4). In the above figure, the blue bars represent the reference standard BA values, whereas the yellow bars indicate the values predicted by the deep neural network. When we compared the performance of the deep neural network model with that of the reference standard, the MSE between the predicted and reference standard BAs was 0.55 (range of estimation errors: -0.5 to 1.1) years for boys and 0.59 (range of estimation errors: -0.8 to 1.2) years for girls (Table 2). These MSE values fall within the range reported in similar studies, where acceptable thresholds for deep learning-based BA assessment models typically range from 0.50 to 0.65 years. This comparison supports the validity of our model’s performance, indicating its reliability for estimating BA in both boys and girls.

**Table 2:** Model Performance Metrics for Boys and Girls

**Metric**	**Boys**	**Girls**
Mean Square Error (MSE)	0.55	0.59
Mean Absolute Error (MAE)	0.59	0.61
Intra-Class Correlation Coefficient (ICC)	0.74	0.82
95% Limits of Agreement (LoA)	-0.8 to 1.2 years	-0.6 to 1.0 years

**Figure 5:**
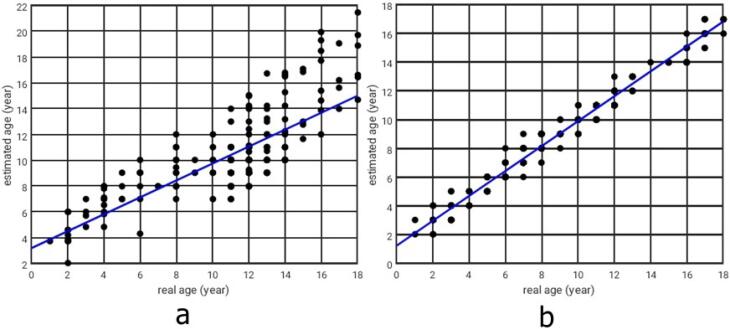
Real Versus Estimated Ages Obtained by the Deep Neural Network (a: for boys, b: for girls)

 In both boys and girls, the estimated BA via the deep neural network model was significantly correlated with the reference BA, with Pearson correlation coefficients of 0.64 for boys (*P* < 0.001) and 0.78 for girls (*P* < 0.001), as presented in Figure 5. The correlation coefficient of 0.64 for boys is considered moderate, while the higher value of 0.78 for girls reflects a stronger predictive capability. These differences may be attributed to gender-specific variations in skeletal development, with potentially greater consistency in the growth patterns observed among girls.

 The model’s performance demonstrated acceptable accuracy, evaluated solely on the 15% validation dataset. For boys, the MSE was 0.55 years, the MAE was 0.59 years, the ICC was 0.74, and the 95% LoA ranged from -0.8 to 1.2 years. For girls, the MSE was 0.59 years, the MAE was 0.61 years, the ICC was 0.82, and the 95% LoA ranged from -0.6 to 1.0 years. These metrics, derived from the validation subset, indicate stronger predictive performance for girls compared to boy.

## Discussion

 Determining BA seems to be an ideal use for AI because it is based on a single standardized non-dominant hand radiograph. Unlike other applications, such as pneumothorax detection, where algorithms may need to assess various aspects of a chest radiograph, BA determination involves only one diagnosis: BA estimation. Many radiologists find this task tedious and time-consuming, requiring a high level of expertise to become proficient. Ensuring reliability and reproducibility is crucial, particularly since sequential examinations are common in clinical practice. Recent advancements in deep learning have marked a new era of research in BA assessment, leading to the development of several AI systems for evaluating BA in several nations. Various algorithms have improved, resulting in faster and more precise predictions.^8,14,16^

 In 2021, Cheng et al utilized an Inception ResNet V2 model to automate BA assessment in a population from Taiwan. The MAD for the study subjects was 0.33 and 0.25 years for the male and female models, respectively.^14^

 In 2021, Wang and colleagues developed an AI system for assessing BA in Tibetan and Han Chinese children. Compared with the experts’ BA assessment, the accuracy of their AI system within 1 year was 84.67% and 89.41%, respectively, and its MAD was 0.65 and 0.56 years, respectively.^17^

 In 2020, Booz and colleagues compared the accuracy of their AI model with that of two pediatric radiologists (as reference standards) in BA assessment. The MAD and root mean square deviation between the AI-derived BA and the reference BA were 0.34 and 0.38 years, respectively.^12^

 In 2018, using cases from Stanford University, Larson and colleagues trained their AI-based BA assessment system. The assessment yielded a root MSE of 0.63 years as a measure of accuracy.^15^

 In 2017, Kim and colleagues introduced a deep learning-based BA estimation system that generated the three most likely BA estimates for a single radiograph. They evaluated the system’s performance via a dataset of 200 cases that were evenly distributed across different age groups from the Asan Medical Center. The study reported a first-rank accuracy of 69.5%.^13^

 Owing to differences in genetic factors and lifestyle patterns, BA assessment systems designed for European and American populations may not be universally applicable to the Asian race. Additionally, numerous studies have highlighted the impact of distinct ethnic backgrounds on bone development and the evaluation of BA.^18^ A comparison of the results of different studies is shown in Table 3.

**Table 3:** Comparison of Previous AI-Based Bone Age Assessment Studies

**Study**	**Methodology**	**Dataset**	**AI Model Used**	**Accuracy**	**Key Findings**
Booz^12^	Compared AI-based BAA with GP method	514 German children (3-17 years)	BoneXpert version 2.1	MAD: 0.34 years (AI) vs. 0.79 years (GP)	AI demonstrated significantly higher accuracy and reduced reading time by 87%
Cheng^14^	Deep learning model (Inception ResNet V2) for automatic bone age assessment	9717 cases from Taiwan	DNN model	MAE: 0.33 years (male), 0.25 years (female)	High accuracy; 99.7% within 2 years of ground truth; model performed better than traditional methods
Wang^17^	AI-based system for BAA applied to Tibetan and Han children	385 children	Deep CNN	Accuracy within 1 year Accuracy: 84.67% (Tibetan), 89.41% (Han)	AI accuracy was lower for 4-6-year-olds due to differences in skeletal maturation
Lee^19^	Fully automated deep learning pipeline for BAA using ImageNet-pretrained CNN	9517 left-hand radiographs	CNN Model	Within 1 year Accuracy: 90.39% (female), 94.18% (male); Within 2 years: 98.11% (female), 99.00% (male)	AI model accurately mimicked human expert evaluation with < 2 s processing time
Larson^15^	Deep learning model compared with expert radiologists	14 036 hand radiographs from Stanford and Colorado hospitals	Deep residual network	MAD: 0.50 years;	AI performance comparable to human experts; MAD slightly better than individual radiologists
Kim^13^	Deep learning-based BAA system evaluated against traditional Greulich-Pyle method	18 940 left-hand radiographs	Deep learning-based AI system	Concordance: 69.5% (first-rank), 86% (top-2), 93% (top-3)	AI-enhanced radiologists’ efficiency, reducing reading time by 18-40% while maintaining high accuracy
Haghnegahdar^20^	Neural network-based BAA using metacarpal and metacarpophalangeal joints dimensions	304 subjects	Neural network	ICC: 0.990 (male), 0.986 (female)	AI-based model showed high agreement with radiologists’ assessment
Dehghani^10^	Computer vision-based BAA using carpal and epiphyseal regions	USC Hand Atlas (training), Iranian dataset (testing)	SVM & KNN	Accuracy: 90% (female), 71.42% (male); MAE: 0.16 (female), 0.42 (male)	AI-based method showed substantial agreement with radiologists, high accuracy for females
Dehghani^11^	AI-based BAA using HOG, LBP, SIFT	442 USC hand radiographs	SVM with 5-fold cross-validation	Accuracy: 73.88% (female), 68.63% (male); MAE: 0.5 years	AI-based approach was reliable and robust for automatic BAA

BAA, Bone Age Assessment; SVM, Support vector Machine; KNN, K-Nearest Neighbors; GP, Greulich-Pyle; CNN, convolutional neural network; HOG, histogram of oriented gradients; LBP, local binary pattern; SIFT, scale-invariant feature transform; MAE, mean absolute error.

 In this study, we evaluated our deep neural network model for 555 Iranian children. To our knowledge, we are the first team to endeavor to develop an AI model for the Iranian pediatric population. Given that males and females typically exhibit varying rates of growth, with boys achieving developmental milestones 12–18 months later than girls do, models designed to differentiate between genders were constructed.^16^

 Our results showed that this AI BA system for Iranian children exhibited reasonable accuracy compared with other models, with MSE values of 0.55 years for boys and 0.59 years for girls. However, the 95% LoA (-0.8 to 1.2 years for boys and -0.6 to 1.0 years for 270 girls) highlight variability in predictions, particularly for boys. This suggests that while the model performs well for girls, additional refinement is necessary to improve reliability for boys and at the extremes of the age range. Furthermore, the moderate correlation coefficient for boys (0.64) underscores the need for improvement. Expanding the training dataset to include more diverse populations and extreme age cases would enhance both the generalizability and clinical applicability of the model.^11,13,14^

 An implication of our deep learning BA model is that it can be used as a diagnostic aid to improve accuracy, reduce interpretation times for radiologists and diminish inter-observer differences.^8^ However, it is important to address potential limitations and biases associated with AI in medical diagnostics to provide a balanced perspective. One significant concern is automation bias, where clinicians may over-rely on AI outputs, even when the model provides inaccurate results. Additionally, the model’s performance depends heavily on the quality and diversity of the training dataset. Since our dataset included only Iranian children, the findings may not generalize well to populations with different genetic, environmental, or lifestyle factors. Furthermore, while the gender-specific analysis highlighted notable differences, the moderate correlation coefficient of 0.64 for boys underscores the need for further refinement to improve reliability across genders. Future work should focus on expanding dataset diversity and addressing these limitations to enhance the clinical applicability of AI models in BA assessment.^17^

 Another thing to note is the observed differences in accuracy between genders, which may come from biological, hormonal, and dataset factors. Females mature earlier and more uniformly due to estrogen, leading to distinct bone growth patterns that AI models learn better from. Males show more variability during puberty, making predictions harder. Moreover, estrogen speeds up female skeletal growth, creating clear milestones, while testosterone causes prolonged and varied male growth. Younger males (4–6 years) show more AI discrepancies due to hormonal variability. Training data often underrepresents male diversity, especially in pre-puberty. Models perform better when male development stabilizes, but female-dominated datasets bias models toward female patterns, increasing male errors. Mitigation strategies, such as incorporating gender-specific features into training, reduce discrepancies. Future AI systems need to combine biological insights and balanced datasets to address these gaps.^10,17,20^

 These issues highlight the need for biologically informed, gender-adaptive AI frameworks for fair diagnostic accuracy.

 Our research provides a foundation for creating deep learning-driven methods for assessing BA that better reflect the current pediatric population and can be customized for particular groups, such as Iranian children. Nevertheless, we intend to perform additional research to explore the application of this method to a larger database.

 Our research has several limitations. First, the test sample size was still relatively small for AI validation, but our patients were somewhat similar to those in some of the previous studies. Additionally, we are the first team that has attempted to develop an AI-based BA assessment model for Iranian children. This study evaluated the performance of the proposed algorithm and revealed that the algorithm is usually capable of correctly estimating BA. Our proposed algorithm can be compared against radiologist assessments as part of this study’s future work.

 Second, the current state of our model may not identify specific conditions that could be detected by a human expert reviewer through image analysis. Examples of such conditions include bone masses, hypochondroplasia, rickets, bone fractures, and congenital syndromes.

 Third, our model was based on comparisons with the clinical assessments of radiologists via the GP atlas. One major criticism regarding the utilization of the GP atlas as a reference standard is that it may not be equally applicable to children from diverse ethnic and racial backgrounds.^18^ However, the primary objective of this study was to assess the feasibility of employing a deep learning model for the automated determination of BA.

## Conclusion

 Our findings demonstrate that the proposed deep learning model achieves reasonable accuracy in BA assessment, with stronger performance in girls compared to boys. However, the relatively wide 95% LoA, particularly for boys, and prediction errors at the extremes of the age range highlight the need for further refinement and validation. While the model shows potential as a supplementary tool for clinicians, future studies should focus on improving prediction accuracy, reducing variability, and validating the model on larger, more diverse datasets before considering widespread clinical implementation. Additionally, addressing edge cases and specific conditions that a human reviewer may detect but the model might overlook, will be essential for enhancing its clinical reliability.
